# Effects of lysine and threonine on milk yield, amino acid metabolism, and fecal microbiota of Yili lactating mares

**DOI:** 10.3389/fvets.2024.1396053

**Published:** 2024-07-03

**Authors:** Jianwei Lin, Hongxin Jing, Jianwen Wang, Jean-Francois Lucien-Cabaraux, Kailun Yang, Wujun Liu, Xiaobin Li

**Affiliations:** ^1^Xinjiang Key Laboratory of Herbivore Nutrition for Meat and Milk, College of Animal Science, Xinjiang Agricultural University, Urumqi, China; ^2^Xinjiang Key Laboratory of Horse Breeding and Exercise Physiology, College of Animal Science, Xinjiang Agricultural University, Urumqi, China; ^3^Faculty of Veterinary Animal Resource Management, Université de Liège, Liège, Belgium

**Keywords:** lactating mare, metabolites, microbiota, lysine, threonine

## Abstract

The nutritional benefits of mare milk are attracting increasing consumer interest. Limited availability due to low yield poses a challenge for widespread adoption. Although lysine and threonine are often used to enhance protein synthesis and muscle mass in horses, their impact on mare milk yield and nutrient composition remains underexplored. This study investigated the effects of lysine and threonine supplementation on 24 healthy Yili mares, mares at day 30 of lactation, over a 120-day period. The mares were divided into control and three experimental groups (six mares each) under pure grazing conditions. The control group received no amino acid supplementation, while experimental groups received varying daily doses of lysine and threonine: Group I (40 g lysine + 20 g threonine), Group II (60 g lysine + 40 g threonine), and Group III (80 g lysine + 60 g threonine). Supplementation in Group II notably increased milk yield, while Groups I and II showed higher milk fat percentages, and all experimental groups exhibited improved milk protein percentages. Additionally, blood levels of total protein, albumin, triglycerides, and glucose were reduced. Detailed analyses from Group II at peak lactation (day 60) included targeted metabolomics and microbial sequencing of milk, blood, and fecal samples. Amino acid metabolomics assessed amino acid content in mare milk and serum, while 16S rRNA gene sequencing evaluated rectal microbial composition. The results indicated that lysine and threonine supplementation significantly increased levels of threonine and creatine in the blood, and lysine, threonine, glutamine, and alanine in mare milk. Microbial analysis revealed a higher prevalence of certain bacterial families and genera, including Prevotellaceae, p_251_o5, and Rikenellaceae at the family level, and *unclassified_p_*251*_o5, Prevotellaceae_UCG*_001, and *Rikenellaceae*_RC9_*gut_group* at the genus level. Multi-omics analysis showed positive correlations between specific fecal genera and amino acids in mare milk. For instance, *Prevotellaceae_UCG_*003, *unclassified Bacteroidetes_*BS11*_gut_group*, and *Corynebacterium* were positively correlated with lysine, while *unclassified Prevotellaceae* was positively correlated with alanine and threonine, and *Unclassified_Bacteroidales_BS11_gut_group* was positively correlated with glutamine. In summary, lysine and threonine supplementation in grazing lactating mares enhanced milk production and improved milk protein and fat quality. It is recommended that herders, veterinarians, and technicians consider amino acid content in the diet of lactating mares. The optimal supplementation levels under grazing conditions for Yili horses were determined to be 60 g lysine and 40 g threonine per day. Future research should explore the molecular mechanisms by which these amino acids influence milk protein and lipid synthesis in mare mammary epithelial cells.

## 1 Introduction

Mare milk is a valuable source of high-quality protein and fatty acids in the human diet, particularly beneficial for children and the elderly (1). The nutritional value of mare milk has been increasingly recognized worldwide, with increasing consumption in countries such as China, Japan, Russia, the Netherlands, and other European nations (2, 3). Today, there is a growing awareness of the excellent physicochemical properties, nutritional value and health potential of mare's milk and its products (4). Amino acids are crucial for mare milk synthesis and are key nutrients for the mare. Lysine and threonine are the most limiting amino acids in horses (5, 6). Supplementation of these amino acids to the lactating mares could significantly enhance mare milk production and composition (7). Despite this, limited research has explored the specific effects of lysine and threonine on mare milk protein anabolism. Investigating the effects of lysine and threonine supplementation on the nutrient composition of mare milk is thus a worthwhile endeavor.

Protein is the primary component of mare milk and a critical economic factor for milk producers (8). The non-protein nitrogen (NPN) content of mare milk is 0.031% (9), which is a surprisingly higher proportion than that in other lactating livestock. The NPN fraction of mare milk contains significant amounts of free amino acids (10). More than 90% of the amino acids in milk are synthesized *de novo* by the mammary glands through uptake from the blood, with the remaining 10% acquired through the direct transport of small peptides, low-molecular-weight proteins, and other immunoreactive proteins (11, 12). Lysine and threonine are the first and second limiting amino acids in the horse, so lysine and threonine supplementation plays an important role in improving milk production and milk composition in mares. However, little research has been conducted on the effects of lysine and threonine on equine milk protein anabolism. Therefore, it is worthwhile to investigate the effects of lysine and threonine supplementation on the nutrients associated with equine milk (5, 13).

Unlike ruminants, where microbial fermentation primarily occurs in the rumen, in equids, it primarily occurs in the large intestine (cecum and colon). Furthermore, 60%−70% of equids' body energy comes from volatile fatty acids (VFAs) produced by microbial fermentation of carbohydrates in the large intestine (14, 15). Colonic microorganisms not only supply energy but also affect the nutritional metabolism of milk (16). Amino acids, the main precursors of milk protein synthesis, play a role in milk protein and lipid synthesis (17, 18). Microbial proteins also play an important role in host protein nutrition and amino acid metabolism (19). Donkeys' large intestine enriches amino acid metabolism through increased expression of colonic transporter proteins and digestive enzymes, regulated by microbial metabolism (20). Lysine and threonine supplementation could influence mare colonic microbial protein synthesis and fermentation products, thereby altering milk composition and nutritional value.

To summarize, the specific effects of lysine and threonine, which are amino acids indispensable for equine health, on the homeostasis of amino acids in the bloodstream and the biochemical profile of mare's milk remain to be elucidated. Additionally, the influence of dietary supplementation of these amino acids on the biosynthesis and enhancement of proteinaceous constituents in mare milk, potentially through modulations in amino acid anabolism and fermentative processes within the intestinal ecosystem, has not been conclusively determined in previous studies. Consequently, the present investigation was conducted to systematically examine the effects of lysine and threonine supplementation on the nutritional and amino acid composition of mare milk, employing targeted metabolomics analyses. The study further characterized the structural and functional diversity of the colonic microbiota through high-resolution 16S *rRNA* gene sequencing and evaluated the metabolites of microbial fermentation using gas chromatography–mass spectrometry techniques.

## 2 Materials and methods

### 2.1 Ethical considerations

The Laboratory Animal Welfare Ethics Committee of Xinjiang Agricultural University approved the animal protocols (2022020) for this study. Research was conducted at Zhaosu Horse Farm, located in Yili Kazakh Autonomous Prefecture, Xinjiang, China.

### 2.2 Animals and experimental design

Twenty-four healthy lactating Yili mares, aged 7–9 years (±1.12), producing 4–5 litters (±0.86) and averaging 428 kg (±33.42) in body weight, day 30 of lactation, were evenly allocated to four groups. The control group received no amino acid supplementation, while the three experimental groups were supplemented with different combinations of lysine and threonine (in powder form) added to their feed bags daily at 09:00 a.m. The supplementation regimen was based on the National Research Council (21) guidelines for lactating mares weighing 400 kg, adjusted to the mares' actual needs to establish a supplementation gradient (22, 23). The specific dosages for each group are detailed in Table 1, sourced from Xinjiang Sesame Trading Company. Mares were manually milked every 2 h from 09:00 a.m. to 05:00 p.m., at 11:00 a.m., 01:00 p.m., 03:00 p.m., and 05:00 p.m. The rest of the day, the mares grazed freely and had access to water. During the 8-h milking period, foals were kept separately from the mares but were allowed to graze together after milking. At the end of the feeding trial, a detailed analysis was conducted. Milk and blood samples from the control group and group II were selected for amino acid metabolome assays, and rectal fecal samples were used for fecal microbial diversity assays, based on milk production, milk composition, and blood biochemical indices of lactating mares.

**Table 1:** Lysine and threonine supplemental feeding rate (g/d-horse).

**Item**	**Control group**	**Group I**	**Group II**	**Group III**
Lysine (g/d·horse)	0	40	60	80
Threonin (g/d·horse)	0	20	40	60

### 2.3 Feeding and management

Foals were separated from their mothers during the 8-h milking period but grazed together for the remaining 16 h of the day. Each morning at 8:30 a.m., mares and foals were moved from the grazing pasture to the milking area. By 9:00 a.m., the experimental group mares received their lysine and threonine-supplemented feed, and foals were separated for milking. At 5:00 p.m., after the final milking session, mares and foals were reunited and returned to the pasture, allowing the mares to graze and drink water freely until the next milking session.

### 2.4 Sample collection

Forage samples were collected from grazing paddocks every 10 days using the flex-cage method (24). Five 2 m × 2 m cages were strategically placed along the feeding routes to ensure even vegetation distribution. After 24 h, fresh grass within these cages was compared with that outside, and selective fresh grass samples were harvested, cut to a uniform height, and collected. A 500-g sample of this grass was then dried in a cool, ventilated area and ground for preservation until further analysis.

Throughout the experiment, 24 mares were manually milked every 2 h over an 8-h period, aligned with the herdsmen's schedule. Horse milk samples were collected at four intervals (11:00 a.m., 01:00 p.m., 03:00 p.m., and 05:00 p.m.) every 10 days, then mixed. Before analyzing the milk composition, the Lactocan Multifunctional Milk Analyser (Europe, model: Lactoscan SP) was calibrated and preheated for 20 min for stabilization, followed by a thorough rinse with distilled water. Mare milk samples were then poured into test cups for analysis. On day 60, at peak lactation, a combined sample prepared in the same ratio was flash-frozen in liquid nitrogen and stored at −80°C for further metabolomics analysis using ultra-high-performance liquid chromatography coupled to tandem mass spectrometry (UHPLC-MS/MS).

On day 60, blood samples were drawn from the jugular vein of mares before morning feeding and collected in two sodium heparin blood collection tubes. Each blood sample (5 mL) was centrifuged at 2,561 g for 15 min to prepare plasma. One plasma tube was analyzed using a fully automated biochemistry analyzer (Shenzhen Myriad BioMedical Electronics Co., Ltd., model: BS-240VET) with kits from the same company, following the instruction manual. Detection parameters included total protein, albumin, triglyceride, and glucose. The other plasma sample was snap-frozen with liquid nitrogen and stored at −80°C for further metabolomics analysis by UHPLC-MS/MS.

Fecal samples, collected every 30 days using the rectal sampling method before the morning supplemental feed, were homogenized, and 50 g of the mixture was flash-frozen in liquid nitrogen and stored at −80°C. Gas chromatography measured VFAs, including acetic, propionic, butyric, isobutyric, valeric, and isovaleric acids (25). An additional 10 g of the sample was reserved for microbiome analysis under the same storage conditions.

### 2.5 Sample determination

#### 2.5.1 Methods of dietary nutrient analysis

Forage samples were dried in an oven at 65°C for 48 h, then crushed and sieved through a 40-mesh screen. Duplicate chemical analyses were conducted. Dry matter, organic matter, and crude protein were analyzed using AOAC methods 930.15, 920.39, and 990.03, respectively (26). Gross energy was measured with an OR2014 calorimeter, a highly precise and intelligent device (Shanghai Orui Instruments & Equipment Co., Ltd., Shanghai, China). Crude fat (EE) was determined via Soxhlet extraction, and crude fiber (CF) was measured through filtration. Calcium (Ca) and phosphorus (P) were analyzed using inductively coupled plasma spectrometry, according to AOAC method 985.01 (2007) (26). The nutritional values of the forages are presented in Table 2.

**Table 2:** Nutrients in pasture^1^.

**Item**	**June (30 d)**	**July (60 d)**	**August (90 d)**	**September (120 d)**
Dry matter DM (%)	89.75	92.82	91.56	88.93
Organic substance OM (%)	91.80	93.41	92.63	90.97
Total energy GE (MJ/kg)	17.43	18.14	17.64	16.98
Crude protein CP (%)	11.36	10.98	10.12	9.64
Crude fat EE (%)	2.70	2.80	2.77	2.66
Crude fiber CF (%)	35.57	36.46	36.75	37.84
Ca (%)	0.75	0.80	0.85	0.90
P (%)	0.14	0.17	0.15	0.14

Feed samples were collected every 10 days and forage samples were collected three times a month, dried, crushed and mixed for nutrient content determination. ^1^The nutrient content is the actual measured value.

#### 2.5.2 Mare milk and blood metabolomics analyses

##### 2.5.2.1 Sample processing

The sample preparation procedure began with adding water to the samples, followed by vigorous vortexing. Next, 50 μL of this mixture was combined with 200 μL of a 1:1 acetonitrile/methanol solution containing mixed ISs, and vortexed again. The samples were then chilled on ice for 30 min and centrifuged at 13,400 g for 10 min.

##### 2.5.2.2 Instrument model

An ultra-high-performance liquid chromatography system coupled with tandem mass spectrometry (UHPLC-MS/MS) (ExionLC™ AD UHPLC-QTRAP^®^ 6500+, AB SCIEX Corp., Boston, MA, USA) was used for analysis.

##### 2.5.2.3 Materials and reagents

After thawing the milk samples on ice, they were well vortexed and diluted with water. Subsequently, 50 μL of this mixture was combined with 200 μL of a 1:1 acetonitrile/methanol solution containing mixed ISs, followed by thorough vortexing. The mixture was chilled on ice for 30 min and then centrifuged at 13,400 g for 10 min. The supernatant was collected for analysis using the LC-MS/MS system. An ultra-high-performance liquid chromatography coupled with tandem mass spectrometry (UHPLC-MS/MS) system (ExionLC™ AD UHPLC-QTRAP 6500+, AB SCIEX Corp., Boston, MA, USA) was employed for amino acid quantification. Chromatographic separation was performed on an ACQUITY UPLC BEH Amide column (2.1 × 100 mm, 1.7 μm) at 50°C. The mobile phase consisted of 0.1% formic acid in 5 mM ammonium acetate (solvent A) and 0.1% formic acid in acetonitrile (solvent B), with a flow rate of 0.30 mL**/**min. The solvent gradient started at 80% B, decreased to 70% B over 2 min, then to 45% B over the next 4 min, before returning to 80% B for the final 2.99 min. The mass spectrometer operated in positive multiple reaction mode, with the following parameters: IonSpray voltage (5500 V), curtain gas (35 psi), ion source temperature (550°C), and ion source gases 1 and 2 (50 and 60 psi). All 23 amino acid standards and 2 stable isotope-labeled standards were sourced from Sigma-Aldrich (St. Louis, MO, USA). Ammonium acetate was also procured from Sigma-Aldrich at analytical grade. Methanol (Optima LC-MS), acetonitrile (Optima LC-MS), and formic acid (Optima LC-MS) were purchased from Thermo Fisher Scientific (Fair Lawn, NJ, USA). Ultrapure water was obtained from Millipore (MA, USA).

The prepared samples underwent targeted metabolomics analysis using LC-MS technology on the SCIEX QTRAP 6500+ mass spectrometry platform. A multi-response monitoring model from the Novogene database facilitated the analysis. Quantification of compounds was achieved using the product ion (Q3), while qualitative analysis utilized the parent ion (Q1). Metabolite identification depended on Q1, Q3, retention time, declustering potential, and collision energy parameters. The limit of quantification was defined by the signal-to-noise ratio method, setting it at an S/N ratio of 10:1. Chromatographic peak areas indicated relative substance quantities, and these areas were analyzed for qualitative and quantitative metabolite information (27). Metabolite annotations were performed using the KEGG database (https://www.genome.jp/kegg/pathway.html), with KEGG annotations plotted using R software (R-3.4.3).

#### 2.5.3 Determination of pH

The pH of the rectum contents was measured with a calibrated portable pH meter (FiveEasy22Meter, Mettler-Toledo International Trading, Shanghai, China) with an accuracy of 0.01. The sample was homogenized and filtered through four layers of gauze for analysis.

#### 2.5.4 High-throughput 16S ribosomal RNA gene sequencing

Total genomic DNA was extracted from feces samples using the TGuide S96 Magnetic Soil/Stool DNA Kit (Tiangen Biotech, Beijing, China) following the manufacturer's instructions. The quality and quantity of the extracted DNA were assessed using electrophoresis on a 1.8% agarosegel, and DNA concentration and purity were determined with a NanoDrop 2000 UV-Vis spectrophotometer (Thermo Scientific, Wilmington, USA). The hypervariable V3-V4 region of the bacterial 16S rRNA gene was amplified with primer pairs 338F: 5′-ACTCCTACGGGAGGCAGCA-3′ and 806R: 5′-GGACTACHVGGGTWTCTAAT-3′. Both forward and reverse 16S primers were tailed with sample-specific Illumina index sequences for deep sequencing. The PCR was performed in a total reaction volume of 10 μL: DNA template 5–50 ng, forward primer (10 μM) 0.3 μL, reverse primer (10 μM) 0.3 μL, KOD FX Neo Buffer 5 μL, dNTP (2 mM each) 2 μL, KOD FX Neo 0.2 μL, and ddH_2_O up to 10 μL. The thermal cycling conditions were: initial denaturation at 95°C for 5 min, followed by 20 cycles of denaturation at 95°C for 30 seconds, annealing at 50°C for 30 seconds, and extension at 72°C for 40 seconds, with a final extension at 72°C for 7 min. The amplified products were purified using the Omega DNA purification kit (Omega Inc., Norcross, GA, USA) and quantified with a Qsep-400 (BiOptic, Inc., New Taipei City, Taiwan, ROC). The amplicon library was paired-end sequenced (2 × 250) on an Illumina NovaSeq 6000 (Beijing Biomarker Technologies Co., Ltd., Beijing, China).

#### 2.5.5 Bioinformatic analysis

Clean reads were classified into amplicon sequence variants (ASVs) using dada2 (28), with ASVs counts < 2 filtered out. Taxonomy annotation of OTUs was performed using the Naive Bayes classifier in QIIME2 (29) with the SILVA database (30) (release 138.1) and a confidence threshold of 70%. Alpha diversity was calculated and displayed using QIIME2 and R software. Beta diversity, which evaluates the similarity of microbial communities from different samples, was determined using QIIME. Principal coordinate analysis (PCoA) was conducted to analyze beta diversity. Linear Discriminant Analysis (LDA) effect size [LEfSe (31)] was employed to test significant taxonomic differences among groups, with a logarithmic LDA score threshold of 4.0 for discriminative features. Redundancy analysis (RDA) was performed in R using the 'vegan' package to explore microbiome dissimilarities among different factors. The sequencing data were analyzed using the online platform BMKCloud (https://www.biocloud.net).

### 2.6 Statistical analysis

Two-factor analysis of variance was conducted using SAS software (version 9.4) to assess differences in lactation performance, blood biochemical indices, and hindgut fermentation parameters between the control and experimental groups. Metabolome principal component analysis (PCA) utilized the R package ggord() to extract peaks from all experimental samples for PCA. Differential metabolites were identified based on Fold Change (FC) and *P-*value, with thresholds set at FC > 1.2 or FC < 0.833 and *P*-value < 0.05. Amino acid levels in blood and milk samples were analyzed using independent-samples *t*-tests. The alpha diversity indices (Chao 1 and Shannon) of the bacterial community were compared between groups using the Wilcoxon signed-rank test. Beta diversity was evaluated using Bray-Curtis dissimilarity and visualized with principal coordinate analysis (PCoA). Spearman correlation analysis in R (version 4.02) assessed correlations between significantly different bacterial genera and milk metabolites in the hindgut of lactating mares, with *P* ≤ 0.05 considered statistically significant.

## 3 Results

### 3.1 Lactation performance and milk composition

The lactation performance results, presented in Table 3, show that the milk yield of experimental group II was significantly higher than that of the control group (*P* < 0.05). Both experimental groups I and II had significantly higher milk fat rates compared to the control group (*P* < 0.05). Additionally, the milk protein rate increased significantly in all experimental groups compared to the control group (*P* < 0.05). However, lactose, non-fat solids, and density did not differ significantly between groups, despite being higher in all experimental groups than in the control group (*P* > 0.05).

**Table 3:** Effects of lysine and threonine supplementation on mare lactation performance and milk composition.

**Item**	**Control group**	**Group I**	**Group II**	**Group III**	**SEM**	***P*** **-value**		
						**T**	**Time**	**T** ^*^ **Time**
Yiel (kg/d)	3.52^b^	3.83^ab^	4.03^a^	3.71^ab^	0.11	< 0.0001	< 0.0001	0.9968
Fat (%)	1.49^b^	1.62^a^	1.69^a^	1.60^ab^	0.04	0.0066	< 0.0001	0.9986
Protein (%)	1.77^b^	1.82^a^	1.84^a^	1.83^a^	0.01	< 0.0001	< 0.0001	0.5177
Lactose (%)	6.35	6.40	6.41	6.41	0.01	0.0049	< 0.0001	0.5617
Non-fat solids (%)	8.96	9.06	9.07	9.07	0.02	0.0017	< 0.0001	0.1020
Density (kg/L)	1.0327	1.0330	1.0330	1.0330	0.00	0.0019	< 0.0001	0.1585

Group I: supplementation with lysine 40 g/d-horse + threonine 20 g/d-horse; Group II: supplementation with lysine 60 g/d-horse + threonine 40 g/horse; Group III: supplementation with lysine 80 g/d-horse + threonine 60 g/horse; T, treatment; Time, Time; SEM, standard error of the mean; ^a, b^Values within a row with different superscripts differ significantly at *P* < 0.05.

### 3.2 Effect of amino acid supplementation on blood biochemical parameters in lactating mares

Changes in blood biochemical parameters after lysine and threonine supplementation are shown in Table 4. Supplementation under grazing conditions resulted in a significant reduction in total protein levels by 9.06, 6.35, and 5.92% in the experimental groups (*P* < 0.01). Albumin levels were also significantly lower by 13.86, 9.50, and 12.10%, respectively, compared to the control group (*P* < 0.01). No significant differences were observed in glucose levels between the groups (P > 0.05). Triglyceride levels were 29.41% (*P* < 0.01), 17.65% (*P* > 0.05), and 23.53% (P < 0.01) higher in the experimental groups.

**Table 4:** Effect of amino acid supplementation on blood biochemical parameters in lactating mares.

**Item**	**Control group**	**Group I**	**Group II**	**Group III**	**SEM**	***P*** **-value**		
						**T**	**Time**	**T** ^*^ **Time**
TP (g/L)	72.48^Aa^	65.91^Bb^	67.88^Bb^	68.19^Bb^	1.00	0.0001	< 0.001	0.0021
ALB (g/L)	29.59^Aa^	25.49^Bb^	26.78^Bb^	26.01^Bb^	0.54	< 0.0001	< 0.0001	0.0107
GLU (mmol/L)	4.30	4.12	4.02	4.29	0.11	0.2299	< 0.0001	0.0430
TG (mmol/L)	0.17^Aa^	0.12^Bb^	0.14^ABab^	0.13^Bb^	0.01	0.0043	< 0.0001	0.0090

Group I: supplementation with lysine 40 g/d-horse + threonine 20 g/d-horse; Group II: supplementation with lysine 60 g/d-horse + threonine 40 g/horse; Group III: supplementation with lysine 80 g/d-horse + threonine 60 g/horse; T: treatment; Time: Time; SEM: standard error of the mean; ^a, b^Values within a row with different superscripts differ significantly at *P* < 0.05; ^A, B^differences are highly significant *P* < 0.01.

### 3.3 Blood amino acid profiling analysis

**Figure 1:**
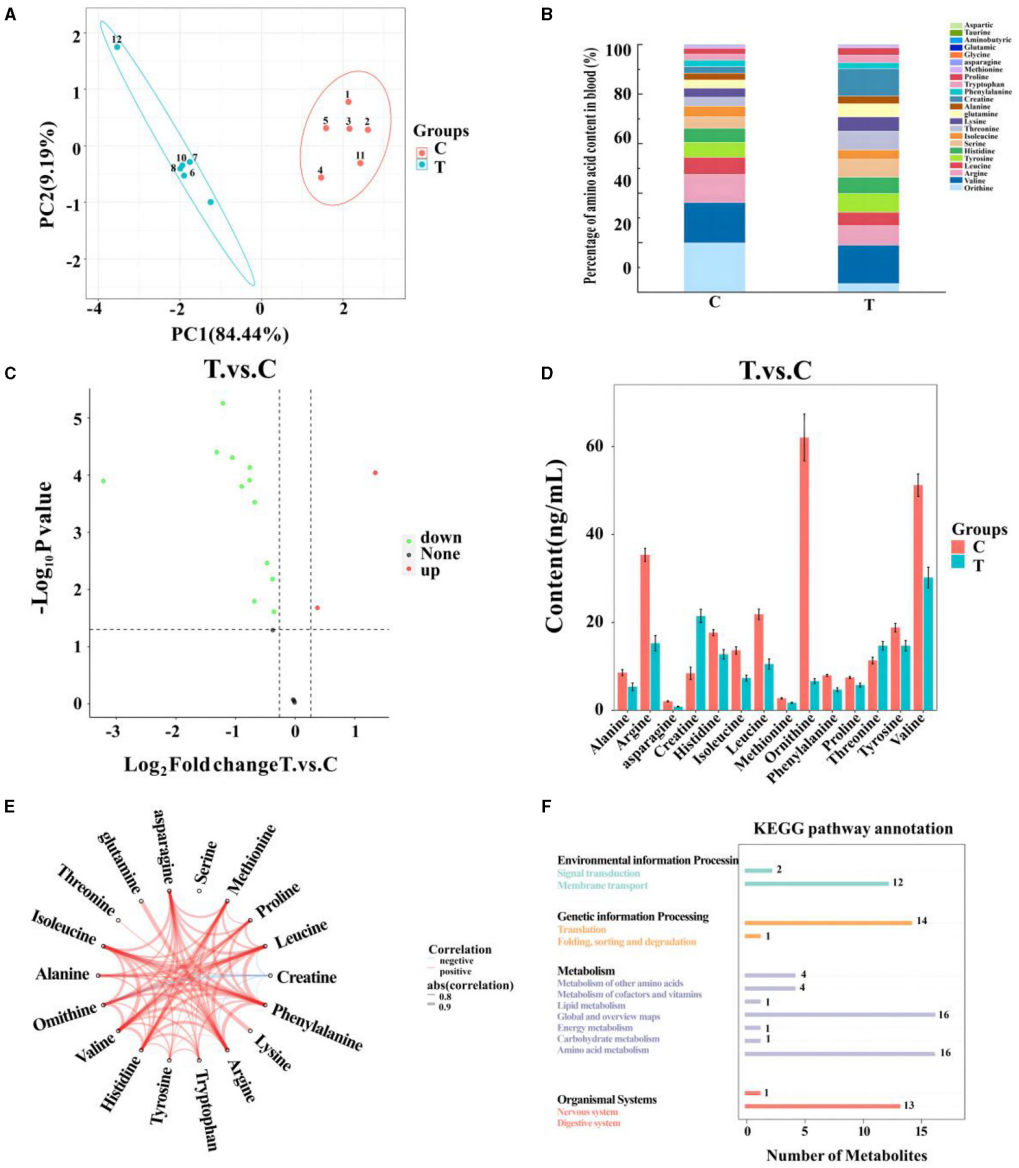
Metabolomics-based analysis of differences in amino acid metabolism in serum of mares supplemented with lysine and threonine. **(A)** Principal component analysis; **(B)** percentage stacking of amino acid content in serum; **(C)** volcano plot; **(D)** histogram with significant differences of amino acid content in mare serum; **(E)** chordal plots reacting to correlations between samples; **(F)** Kyoto Encyclopedia of genes and genomes pathway map percentage stacking plot; C, Control; T, treated group.

After quality control, 18 amino acids detected in mare blood were quantified, with 14 showing significant variation (Table 5). Differences between the control and experimental groups were determined using PCA (Figure 1A). Each amino acid's contribution was depicted as a percentage of the total 23 amino acids in the serum (Figure 1B). The distribution of metabolites was illustrated using a volcano plot (Figure 1C), where the significance of the 18 amino acids was assessed, revealing two significantly upregulated amino acids (creatine and threonine) and 12 significantly downregulated amino acids. These 14 amino acids were displayed in a histogram to detail their concentrations (Figure 1D). The chord plot illustrated the correlations among the 18 detected amino acids, highlighting a negative correlation between ornithine and creatine (Figure 1E). KEGG pathway analysis revealed the roles of these amino acids in lactating mare blood, primarily in membrane transport, translation, nucleotide metabolism, and amino acid synthesis and metabolism (Figure 1F).

**Table 5:** Quantification of amino acids in mare blood (μg/mL).

**Categorization**	**Item**	**Control group**	**Group II**	**SEM**	***P*-value**
Essential amino acid	Methionine	2.74^A^	1.71^B^	0.19	0.00
	Leucine	21.84^A^	10.53^B^	1.67	0.00
	Phenylalanine	7.97^A^	4.71^B^	0.51	0.00
	Lysine	11.3	11.23	0.92	0.94
	Argine	35.37^A^	15.28^B^	2.31	0.00
	Tryptophan	7.57^a^	5.84^b^	0.78	0.05
	Histidine	17.68^A^	12.76^B^	1.29	0.00
	Valine	51.22^A^	30.21^B^	3.47	0.00
	Isoleucine	13.63^a^	7.32^b^	1.07	0.00
	Threonine	11.34^b^	14.70^a^	1.23	0.02
Non-essential amino acids	Serine	14.43	14.1	1.60	0.84
	Proline	7.47^A^	5.75^B^	0.51	0.01
	Creatine	8.45^B^	21.47^A^	2.07	0.00
	Tyrosine	18.8^a^	14.7^b^	1.55	0.02
	Ornithine	62.05^A^	6.68^B^	5.34	0.00
	Alanine	8.59^a^	5.35^b^	1.12	0.02
	Glutamine	10.51	10.38	0.84	0.88
	Asparagine	2.07^A^	0.83^B^	0.18	0.00

SEM, standard error of the mean; ^a, b^Values within a row with different superscripts differ significantly at *P* < 0.05; ^A, B^differences are highly significant *P* < 0.01.

### 3.4 Metabolomics analysis of amino acids in mare milk

**Figure 2:**
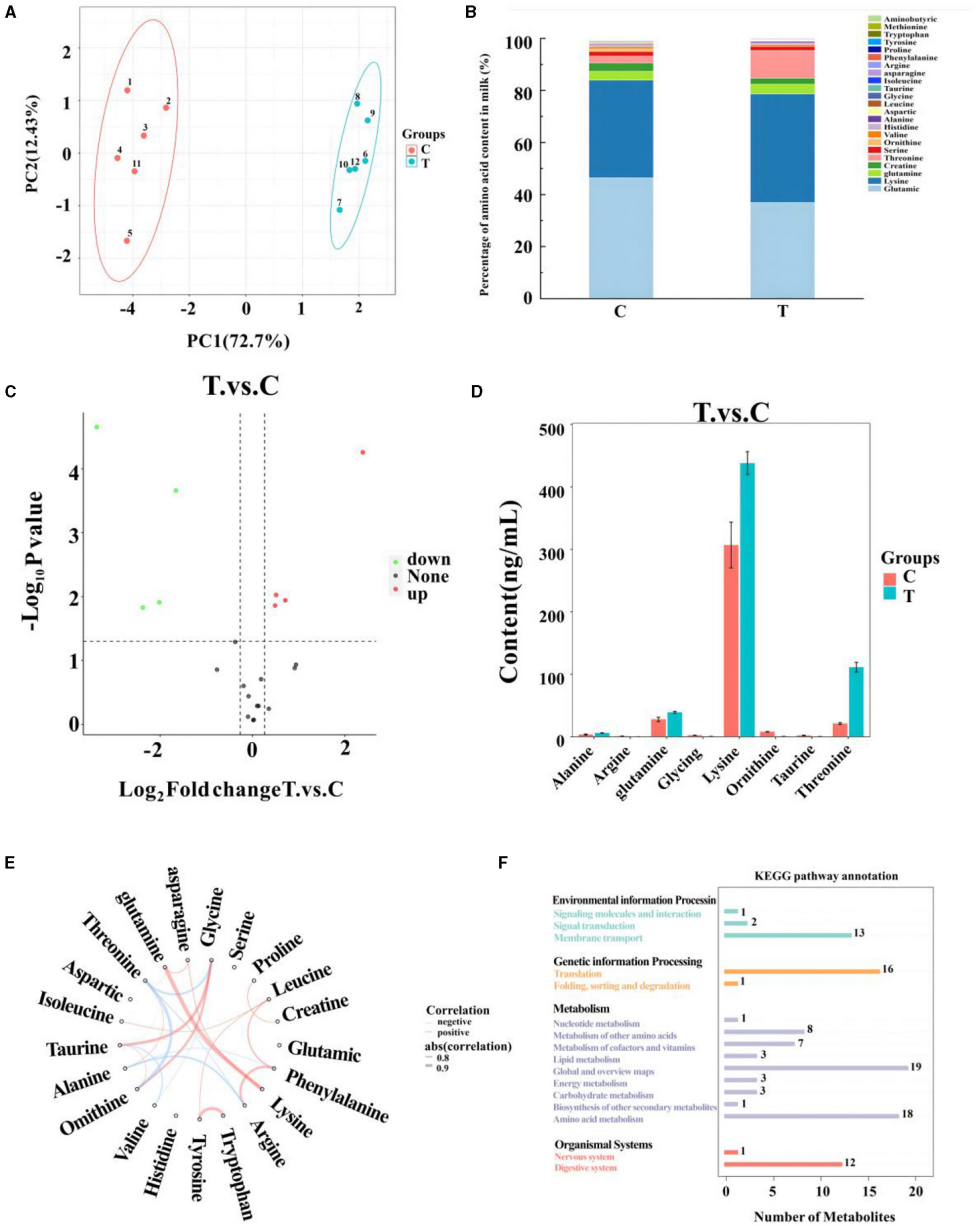
Metabolomics-based analysis of differential metabolism of amino in milk of mares supplemented with Lysine and Threonine. **(A)** Principal component analysis; **(B)** percentage stacking of amino acid content in milk; **(C)** volcano plot; **(D)** histogram with significant differences of amino acid content in mare serum milk; **(E)** chordal plots reacting to correlations between samples; **(F)** Kyoto encyclopedia of genes and genomes pathway map percentage stacking plot; C, Control; T, treated group.

A total of 21 amino acids were quantified in mare milk after quality control measures, with eight showing significant differences: arginine, glycine, ornithine, taurine, alanine, glutamine, lysine, and threonine (Table 6). PCA (Figure 2A) highlighted differences between the control and experimental groups. Each amino acid's proportionate size, representing its percentage of the 23 amino acid contents in the milk, was illustrated (Figure 2B). A volcano plot showed the overall distribution of metabolites (Figure 2C), identifying four significantly downregulated amino acids (arginine, glycine, ornithine, and taurine) and four significantly upregulated amino acids (alanine, glutamine, lysine, and threonine) in the experimental group. Histograms detailed the content variations of these eight amino acids (Figure 2D). The chord plot revealed correlations among the 21 amino acids detected in milk, highlighting relationships such as glycine with taurine and ornithine, phenylalanine with arginine, and tryptophan with tyrosine (Figure 2E). KEGG pathway analysis indicated the roles of these amino acids in lactating mare milk, focusing on membrane transport, translation, nucleotide metabolism, and amino acid metabolism (Figure 2F).

**Table 6:** Quantification of amino acids in mare milk (μg/mL).

**Categorization**	**Item**	**Control group**	**Group II**	**SEM**	***P*-value**
Essential amino acid	Leucine	2.63	2.46	0.53	0.76
	Phenylalanine	0.76	0.44	0.19	0.14
	Lysine	306.91^B^	438.11^A^	40.95	0.01
	Argine	1.10^A^	0.21^B^	0.26	0.01
	Tryptophan	0.06	0.12	0.03	0.12
	Histidine	6.57	5.75	0.67	0.25
	Valine	6.89	7.85	0.66	0.18
	Isoleucine	1.93	2.07	0.21	0.51
	Threonine	21.26^B^	111.45^A^	7.89	0.00
Non-essential amino acids	Glycine	2.42^A^	0.77^B^	0.29	0.00
	Serine	14.88	16.32	2.13	0.52
	Proline	0.27	0.21	0.03	0.05
	Creatine	25.98	24.53	1.53	0.36
	Glutamic	378.74	386.4	39.01	0.85
	Tyrosine	0.17	0.21	0.08	0.57
	Ornithine	8.03^A^	0.77^B^	0.50	0.00
	Alanine	3.72^B^	6.09^A^	0.77	0.01
	Taurine	2.23^A^	0.55^B^	0.46	0.00
	Aspartic	2.63	2.67	0.19	0.86
	Glutamine	27.92^b^	39.22^a^	3.79	0.01
	Asparagine	1.88	3.55	1.01	0.13

SEM, standard error of the mean; ^a, b^values within a row with different superscripts differ significantly at *P* < 0.05; ^A, B^differences are highly significant *P* < 0.01.

### 3.5 Manure fermentation parameters

Table 7 presents the results of hindgut fermentation parameters, revealing a significantly lower pH in experimental group II compared to the control group (*P* < 0.01). Although the contents of propionic acid, butyric acid, valeric acid, and total volatile fatty acids were lower in group II, the differences were not statistically significant (*P* > 0.05).

**Table 7:** Effect of lysine and threonine on mare fecal fermentation parameters.

**Item**	**Control group**	**Group II**	**SEM**	***P*** **-value**		
				**T**	**Time**	**T** ^*^ **Time**
pH	6.43^A^	6.18^B^	0.04	< 0.0001	0.0093	0.0505
Acetate (mmol/L)	23.36	23.47	1.07	0.9431	< 0.0001	0.0015
Propionate (mmol/L)	3.88	3.81	0.20	0.8030	< 0.0001	0.0008
Butyrate (mmol/L)	1.39	1.21	0.11	0.2665	< 0.0001	0.0041
Isobutyrate (mmol/L)	0.48^A^	0.29^B^	0.03	0.0003	< 0.0001	0.1988
Valerate (mmol/L)	0.58	0.20	0.18	0.1428	0.1456	0.5411
Isovalerate (mmol/L)	0.80^a^	0.55^b^	0.07	0.0226	< 0.0001	0.0418
Total VFA (mmol/L)	30.50	29.55	1.48	0.6514	< 0.0001	0.0035

Group II: supplementation with lysine 60 g/d-horse + threonine 40 g/horse; T, treatment; Time, Time; SEM, standard error of the mean; VFA, Volatile Fatty Acid; ^a, b^values within a row with different superscripts differ significantly at *P* < 0.05; ^A, B^differences are highly significant *P* < 0.01.

### 3.6 Microbial composition of mare hindgut

**Figure 3:**
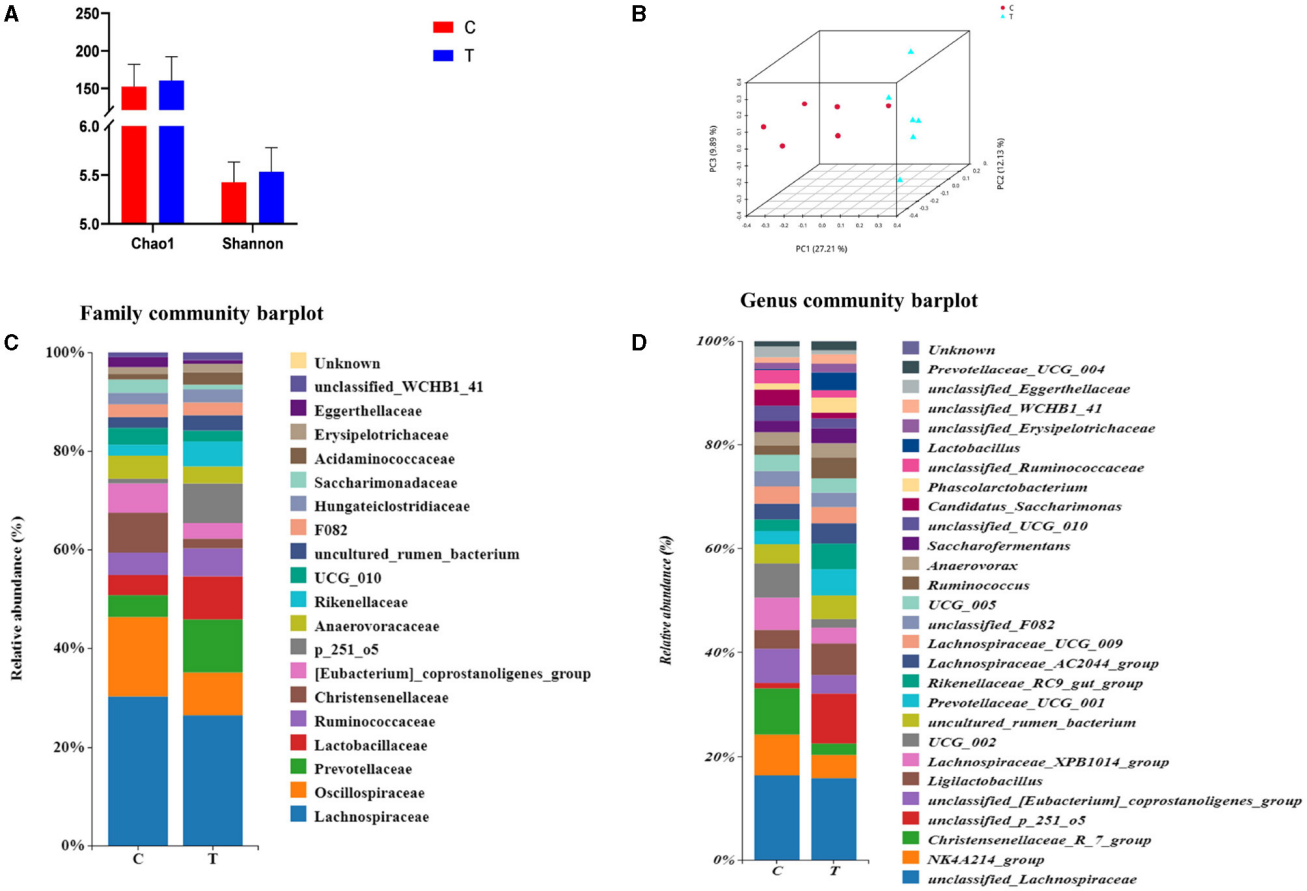
Rectal fecal microbial diversity in lactating mares. **(A)** Changes in the alpha diversity at the genus level; **(B)** changes in the beta diversity at the genus level; **(C)** microbial diversity analysis at the family level >1%; **(D)** microbial diversity analysis at genus level >1%.

**Figure 4:**
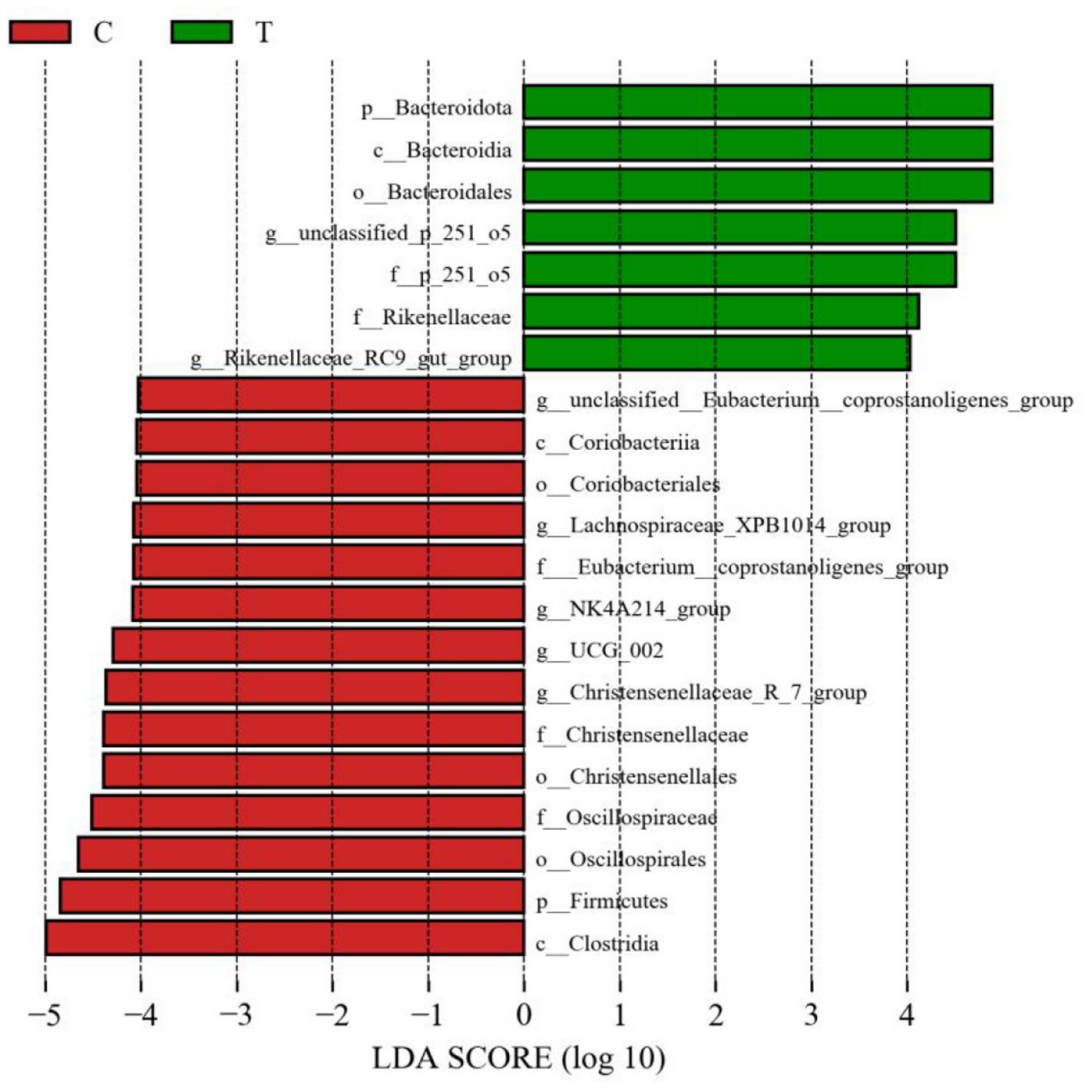
LEfSe analysis of fecal bacteria in the hindgut of mares supplemented with lysine and threonine. Significant differences were tested using linear discriminant analysis effect size (LEfSe) analysis with a linear discriminant analysis score of >4 and a *p-*value of < 0.05.

No significant differences were observed between experimental group II and the control group in terms of the Chao 1 index and Shannon's index (Figure 3A). However, PCoA results indicated significant differences in the bacterial communities in the hindgut of lactating mares between the control and experimental groups (Figure 3B). The experimental group exhibited a higher prevalence of certain bacterial families and genera, including Prevotellaceae, p_251_o5, and Rikenellaceae at the family level (family level > 1%), and *unclassified_p_251_o5, Prevotellaceae_UCG_*001, and *Rikenellaceae_*RC9*_gut_group* at the genus level (genus level > 1%) (Figures 3C, D). LEfSe analysis identified 21 bacterial taxa that differed significantly between the experimental and control groups. The experimental group showed increased relative abundance of *p_251_o5* and *Rikenellaceae* at the family level, and *unclassified _p_251_o5* and *Rikenellaceae_*RC9*_gut_group* at the genus level. Conversely, the *Lachnospiraceae_*XPB1014*_group, UCG_*002, and *Christensenellaceae_*R_7*_group* were more abundant in the control group (Figure 4).

### 3.7 Correlation analysis

**Figure 5:**
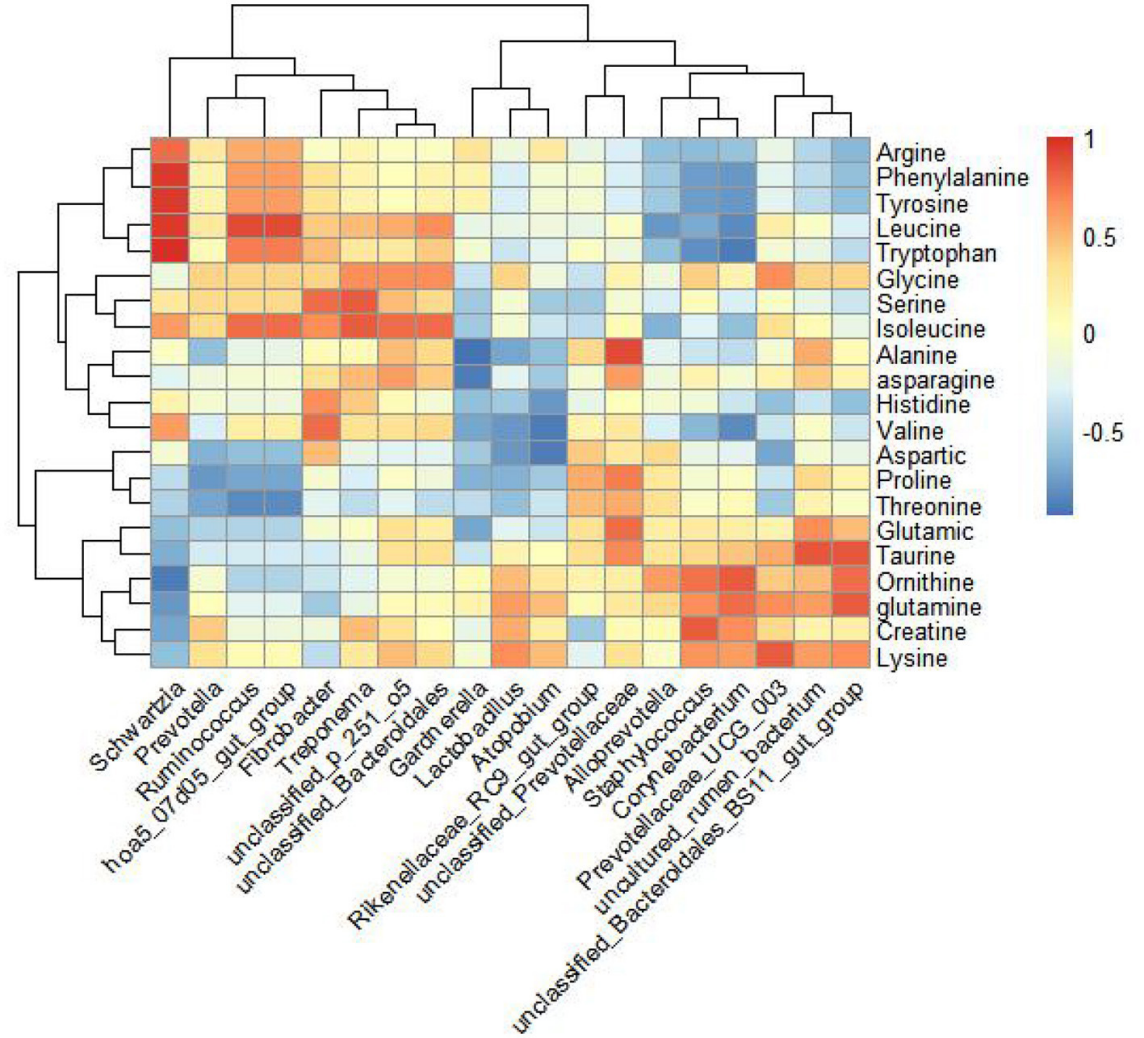
Correlation analysis of amino acids detected by amino acid targeted metabolism in mare's milk and 16S rRNA sequencing genus level differential bacteria in feces. Spearman's correlation analysis of amino acids in genus-level microorganisms and horse milk by selecting genus-level microorganisms and amino acids in horse milk with >3 points of LDA detected in hindgut feces and with colony relative abundance >0.1% of the control differential bacteria.

In the experimental group, a comparative analysis using Spearman's correlation evaluated all differential genera with a relative abundance >0.1%, genera with LDA scores >3, and amino acids detected in milk, as well as horse milk proteins and lipids (Figure 5). The results indicated that the genera *Prevotellaceae_UCG_*003 and *unclassified_Bacteroidales_*BS11*_gut_group* were positively correlated with lysine in milk. *Unclassified_Prevotellaceae* showed a positive correlation with alanine and threonine, while *unclassified_Bacteroidales_*BS11*_gut_group* was positively correlated with glutamine content.

## 4 Discussion

Horse milk, compositionally similar to human milk and hypoallergenic, holds significant value for consumption and research (32, 33). It contains comparable levels of lactose and protein to cow and human milk but offers twice the amount of whey protein and lysozyme, making it beneficial for children allergic to milk protein (1, 34). However, its limited yield and protein content restrict broader usage (35, 36). Both limiting and essential amino acids influence milk production and composition in lactating females (37). Despite being essential for equines (23), lysine and threonine's effects on mare milk composition have not been extensively studied. Milk production is a complex process involving biochemical synthesis of nutrients, primarily through the selective uptake and concentration of plasma by mammary epithelial cells (16, 38). Administering different doses of lysine and threonine to grazing lactating mares enhanced milk production to varying degrees. Specifically, consuming 60 g of lysine and 40 g of threonine significantly increased lactation volume, milk protein rate, and milk fat rate. At peak lactation, nutritional requirements rise sharply as milk production increases. Blood glucose and triglycerides serve as indicators of glucolipid metabolism and energy conversion balance (39). Insufficient dietary energy leads to protein being converted into sugar and fat to maintain metabolic activities. Lactating mares fed lysine and threonine exhibited lower glucose and triglyceride levels than the control group (40). This may be due to insufficient energy intake or amino acid imbalance in the control group, causing protein breakdown into sugars and fats. Lysine and threonine, as limiting amino acids, increase essential amino acid content in the blood and accelerate the nitrogen cycle, thereby boosting milk yield and protein rate (41). Lysine promotes mammary epithelial cell proliferation and regulates milk protein synthesis through the SATA signaling pathway (42). Threonine, with its sweet taste, enhances feed intake and conversion rates, promoting nutrient absorption in the mammary glands and thereby increasing lactation. The lower milk production in test group III compared to group II might result from lysine's stimulating odor affecting appetite (43, 44). Studies suggest lysine and threonine requirements for lactating mares weighing 400 kg range from 54–61.2 g and 33.48–37.94 g per day, respectively (45), aligning with group II's supplementation in this study. Thus, supplementing 60 g of lysine and 40 g of threonine per day is recommended for Yili mares weighing 400 kg.

Mare milk protein, a valuable nutrient, plays a crucial role in assessing milk quality and serves as a significant protein source for humans (46, 47). The production of milk proteins relies heavily on the supply of amino acids in the mammary gland, with over 90% synthesized directly from amino acids absorbed from the bloodstream (12, 13). Metabolic pathway analysis of serum metabolites reveals significant enrichment in processes such as membrane transport, amino acid metabolism, and the digestive system, primarily associated with amino acid metabolism and digestion. These processes are linked to variations in threonine and creatine levels in the plasma. Elevated serum concentrations of essential and total amino acids in the experimental group suggest that the mammary gland not only uses these amino acids for protein synthesis but also employs them as signaling molecules to regulate milk protein synthesis (48–50). Threonine can be converted to 2-amino-3-oxobutyrate through a glycine-dependent pathway and then degraded by 2-amino-3-oxobutyrate coenzyme A ligase into acetyl coenzyme A and glycine. Acetyl coenzyme A enters the tricarboxylic acid cycle, supplying energy for metabolism (51). In the mammary gland, glycine regulates protein synthesis by activating mTORC3 and inhibiting protein hydrolysis via a PI1K/Akt-dependent mechanism. Glycine also aids in damage repair in mammary cells as part of the endogenous antioxidant glutathione (52). Moreover, glycine is closely linked to creatine synthesis. Creatine in the blood regulates feed intake and provides methyl groups during choline degradation to glycine, thereby promoting protein synthesis (53, 54). Higher serum concentrations of threonine and creatine in the experimental group may result from increased ATP utilization for physiological synthesis and metabolism (55).

The study on milk metabolomics revealed notable increases in lysine, threonine, alanine, and glutamine levels following supplementation of lysine and threonine. Metabolic pathway analysis indicated significant involvement of these metabolites in processes such as membrane transport, amino acid transport and metabolism, and the digestive system, likely due to the lysine and threonine supplementation. In diets primarily composed of grass and grains, lysine and threonine are often the first and second limiting amino acids, respectively (5, 56). Lysine plays a crucial role in the development of the mammary gland and the production of milk. It activates the PI3K/PKB signaling pathway upstream of mTOR, affecting mTORC1 activity and leading to the phosphorylation of S6K1 and the activation of the ribosomal S6 protein, thus enhancing mRNA translation and protein synthesis (57–59). This activation aligns with the observed increase in milk protein content. During the post-partum period, mammals experience a surge in milk production and the synthesis of milk protein and fat, coupled with increased intestinal activity. The intestines rely on glutamine as an energy source, helping to preserve glucose (60). Glutamine converts to glutamate through the action of glutaminase and subsequently to α-ketoglutarate via glutamate dehydrogenase or transamination. α-Ketoglutarate may enter the tricarboxylic acid cycle directly or convert into acetyl-CoA by reacting with alanine, providing the necessary substrates and energy for milk protein synthesis (61). Cow milk protein allergies, prevalent in children with rates between 1.9% and 4.9%, typically manifest before the age of one, necessitating hypoallergenic substitutes for breast milk (62). Dietary amino acids play a crucial role in gut health, with the amino acid profile of breast milk influencing the infant's gut exposure. Elevated free threonine in breast milk can bolster infant immune function by reducing the prevalence of *Aspergillus* spp. in the gut (63). Similarly, higher milk glutamine levels enhance intestinal barrier function by improving protease signaling and reducing tight junction protein expression (64). Wang et al. (65) suggested that lysine and glutamine, acting as binding receptors for α-lactalbumin and β-lactoglobulin in cow's milk, may reduce the incidence of allergic diseases in infants. The increased levels of lysine and glutamine in horse milk observed in this study could offer comparable benefits (65). Reche et al. (66) enhanced the nutritional quality of infant formula by adding lysine, threonine, and partially hydrolyzed rice proteins to mimic breast milk's amino acid profile. Their results indicated that the formula did not trigger allergic reactions in infants and was well-tolerated due to its pleasant smell, texture, and taste (66). Ventura et al. found that different amino acids influence the flavor and texture of milk, potentially affecting its quality (67). The increase in lysine, threonine, alanine, and glutamine in this study aligns with Ventura et al.'s findings, suggesting new avenues for developing functional milk products.

Growing studies indicate that the nutrient content of mare milk and volatile fatty acid content in the intestine are closely related to lactation, with volatile fatty acids primarily derived from the fermentation of feed in the cecum and colon (68, 69). In this study, lysine and threonine supplementation did not significantly improve fecal volatile fatty acid content in lactating mares but did enhance milk protein, milk fat, and amino acid content (including arginine, glycine, ornithine, taurine, alanine, glutamine, lysine, and threonine). This may be due to herbivores producing fewer volatile fatty acids through enteric fermentation in the large intestine and volatile fatty acids not being directly involved in milk component synthesis, as described by Hintz et al. (70). Lysine and threonine supplementation significantly increased the levels of several fecal microbial genera, including *Rikenellaceae_*RC9*_gut_group, Prevotellaceae_UCG_*001, *unclassified*_p_251_o5, *Prevotellaceae_UCG_*003, *unclassified_Prevotellaceae*, and *unclassified_Bacteroidales_BS11_gut_group*. This aligns with Wang et al.'s review, which suggests that lysine and threonine in animal diets maintain gut health and improve gut microbiota (71). Changes in the relative abundance of *Prevotellaceae_UCG_*003 correlate with changes in milk fat content in mare milk (69). Our study showed higher relative abundance of these bacteria, consistent with the higher milk fat rate in the test group's milk. Correlation analysis revealed a positive correlation between *Prevotellaceae_UCG_*003 and lysine in milk.

Lysine is involved in lipid metabolism, accelerating fat metabolism in the body (72). It provides structural components for carnitine synthesis, a precursor that helps long-chain fatty acids enter mitochondria for oxidative decomposition, supplying energy and substrates for lactation synthesis (73). Thus, lysine in milk may contribute to milk protein synthesis and affect milk lipid secretion with the help of microorganisms, though the exact mechanism requires further investigation. In addition to lysine, threonine, alanine, and glutamine are involved in milk protein synthesis. Correlation analysis showed that *Unclassified_Prevotellaceae* was positively correlated with alanine and threonine, while *unclassified_Bacteroidales*_BS11_*gut_group* was positively correlated with glutamine. Therefore, *Unclassified_Prevotellaceae* and *unclassified_Bacteroidales_*BS11*_gut_group* may be associated with threonine, alanine, and glutamine in milk protein synthesis, though the exact mechanisms need further exploration.

Nutritional science competence is essential for veterinarians to improve livestock health (74). Despite advancements in veterinary education, understanding the effects of lysine and threonine supplementation on lactating mares remains insufficient. Lysine and threonine supplementation enhances mares' production performance and nutrient absorption in unweaned foals, promoting growth and improving breeding economics. This study provides valuable insights for veterinarians, technicians, and farmers in selecting effective feed additives for mare breeding (6).

## 5 Conclusion

**Figure 6:**
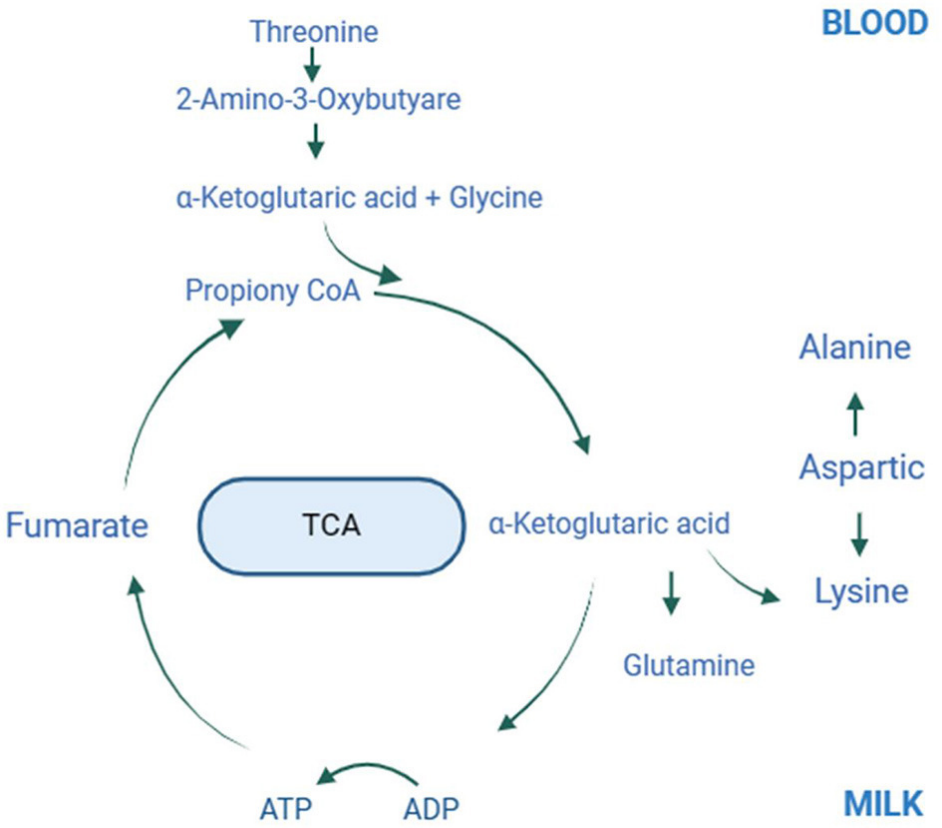
Plot of amino acid metabolism in mare blood and milk after lysine and threonine supplementation feeding. The possible molecular mechanisms involved in the synthesis of lysine, threonine, glycine, alanine and glutamine in milk and blood via the tricarboxylic acid cycle after lysine and threonine supplemental feeding. TCA, Tricarboxylic acid cycle.

Supplementing grazing lactating mares with lysine and threonine increased milk production and improved milk protein and fat quality. Administering 60 g of lysine and 40 g of threonine per horse daily during peak lactation significantly raised threonine and creatine levels in the blood, lowered total protein and albumin levels, and enhanced lysine, threonine, alanine, and glutamine concentrations in the milk, thus increasing its protein content. Figure 6 illustrates the potential molecular mechanisms by which lysine and threonine enhance mare milk protein. Additionally, supplementation increased the abundance of *Prevotellaceae, p_251_o5, Rikenellaceae, Prevotellaceae_UCG_*003, *unclassified_Prevotellaceae, unclassified_p_251_o5, Prevotellaceae_UCG*_001, *unclassified_Bacteroidales_*BS11*_gut_group*, and *Rikenellaceae_*RC9*_gut_group*. Therefore, herders, veterinarians, and technicians should consider the amino acid content in the mare's diet. Under grazing conditions, the recommended supplementation for lactating Yili mares is 60 g/d-horse for lysine and 40 g/d-horse for threonine. Future studies should investigate the molecular mechanisms by which lysine and threonine affect the synthesis of mare milk proteins and lipids by exogenously adding these amino acids to mare mammary epithelial cells.

## Data availability statement

The data presented in the study are deposited in the NCBI Database repository, accession number PRJNA1123219.

## Ethics statement

The animal study was approved by Laboratory Animal Welfare Ethics Committee of Xinjiang Agricultural University. The study was conducted in accordance with the local legislation and institutional requirements.

## Author contributions

JL: Software, Visualization, Writing – original draft. HJ: Validation, Writing – original draft. JW: Funding acquisition, Writing – review & editing. J-FL-C: Funding acquisition, Writing – review & editing. KY: Supervision, Writing – review & editing. WL: Supervision, Writing – review & editing. XL: Funding acquisition, Project administration, Supervision, Writing – review & editing.
